# Characterization and Management of Cytokine Release Syndrome From the MonumenTAL‐1 Study of Talquetamab in Patients With Relapsed/Refractory Multiple Myeloma

**DOI:** 10.1002/cam4.71276

**Published:** 2025-10-02

**Authors:** Niels W. C. J. van de Donk, Ajai Chari, Thomas Martin, Amrita Krishnan, Leo Rasche, Jing Christine Ye, Rakesh Popat, Brea Lipe, Cesar Rodriguez, Carolina Schinke, Sheri Skerget, Deeksha Vishwamitra, Raluca Verona, Jue Gong, Indrajeet Singh, Michela Campagna, Tara Masterson, Brandi Hilder, Jaszianne Tolbert, Thomas Renaud, M. Damiette Smit, Christoph Heuck, Maria‐Victoria Mateos

**Affiliations:** ^1^ Amsterdam University Medical Center Vrije Universiteit Amsterdam Amsterdam the Netherlands; ^2^ University of California San Francisco San Francisco California USA; ^3^ University of California San Francisco, Helen Diller Family Comprehensive Cancer Center San Francisco California USA; ^4^ City of Hope Comprehensive Cancer Center Duarte California USA; ^5^ University Hospital of Würzburg Würzburg Germany; ^6^ MD Anderson Cancer Center University of Texas Houston Texas USA; ^7^ University College London Hospitals, NHS Foundation Trust London UK; ^8^ University of Rochester Medical Center Rochester New York USA; ^9^ Department of Hematology and Oncology, School of Medicine Wake Forest University Winston‐Salem North Carolina USA; ^10^ Myeloma Center University of Arkansas for Medical Sciences Little Rock Arkansas USA; ^11^ Johnson & Johnson Spring House Pennsylvania USA; ^12^ Johnson & Johnson Madrid Spain; ^13^ Johnson & Johnson Raritan New Jersey USA; ^14^ Johnson & Johnson Leiden the Netherlands; ^15^ University Hospital of Salamanca/IBSAL/CIC/CIBERONC Salamanca Spain

**Keywords:** clinical management, cytokine release syndrome, GPRC5D, interleukin‐6, talquetamab, tocilizumab

## Abstract

**Introduction:**

Cytokine release syndrome (CRS) is a common adverse event associated with T‐cell redirection therapies (TCRT), including talquetamab, the first GPRC5D × CD3 bispecific antibody approved for relapsed/refractory multiple myeloma (RRMM). We describe CRS with talquetamab and implications for clinical practice.

**Methods:**

Patients without prior TCRT received talquetamab 0.4 mg/kg weekly (QW) and 0.8 mg/kg every other week (Q2W), with two or three step‐up doses, respectively, plus pretreatment with a glucocorticoid, antihistamine, and antipyretic. A separate cohort of patients with prior TCRT received talquetamab at either the QW or Q2W schedule. CRS was graded per American Society for Transplantation and Cellular Therapy criteria and managed per study protocol.

**Results:**

Across talquetamab QW (*n* = 143), Q2W (*n* = 145), and prior TCRT (*n* = 51) cohorts, most CRS events occurred during step‐up doses and were grade 1 or 2; grade 3 CRS events were rare. Approximately one‐third of patients experienced more than one CRS event. Fewer patients experienced subsequent CRS events if tocilizumab was used versus not used to treat their first CRS event. Overall response rates with talquetamab were similar among patients with and without tocilizumab to manage CRS. Baseline characteristics were not associated with CRS incidence, recurrence, duration, or severity, whereas immune biomarkers showed some trends in association with CRS parameters.

**Conclusion:**

CRS outcomes with talquetamab were consistent with those seen with other TCRT in RRMM, including teclistamab (BCMA×CD3 bispecific antibody), indicating a similar clinical approach, including early vigilance and prompt treatment of CRS.

**Trial Registration:**

NCT03399799/NCT04634552

## 1 Introduction

Cytokine release syndrome (CRS), a condition involving systemic immune activation that can be triggered by infections or certain therapies, is characterized by fever, tachypnea, headache, tachycardia, hypotension, rash, hypoxia, and/or multiorgan system dysfunction [1, 2, 3]. CRS is commonly reported in patients with multiple myeloma treated with T‐cell redirection therapies (TCRT), including chimeric antigen receptor T cell (CAR‐T) therapy and the bispecific antibodies (bsAb) talquetamab, teclistamab, and elranatamab, consistent with the mechanism of action of these drug classes [4, 5, 6, 7, 8, 9, 10, 11]. The American Society for Transplantation and Cellular Therapy (ASTCT) and International Myeloma Working Group (IMWG) have published guidance on managing CRS [3, 12], which often includes the use of tocilizumab (an interleukin‐6 [IL‐6] receptor monoclonal antibody) or dexamethasone, depending on the event severity [3, 7, 13, 14, 15].

Talquetamab is a bsAb targeting G protein–coupled receptor class C group 5 member D (GPRC5D) and CD3 [14, 16, 17, 18]. In the phase 1/2 MonumenTAL‐1 study, talquetamab demonstrated overall response rates (ORRs) of > 71% with the recommended phase 2 doses (RP2Ds) of 0.4 mg/kg weekly (QW) and 0.8 mg/kg every other week (Q2W) and 65% in a separate cohort of patients with prior exposure to TCRT receiving either dose schedule [14, 16]. Talquetamab was well tolerated; CRS was the most frequent adverse event, occurring in 75%–79% of patients [14, 16]. Based on these results, the U.S. Food and Drug Administration (FDA) and European Medicines Agency approved talquetamab for the treatment of patients with relapsed/refractory multiple myeloma (RRMM) who have received at least four and at least three prior lines of therapy (LOTs), respectively, including an immunomodulatory drug (IMiD), proteasome inhibitor (PI), and anti‐CD38 monoclonal antibody [19, 20].

Here, we describe CRS in patients treated with talquetamab to inform clinical practice and applicability of current guidelines for bispecific antibodies in RRMM.

## 2 Materials and Methods

### 2.1 Study Design and Patients

The methodology of MonumenTAL‐1 (NCT03399799/NCT04634552) has been published previously [14, 16]. MonumenTAL‐1 comprised a phase 1 dose escalation/expansion and a phase 2 assessing the efficacy and safety of talquetamab at the 0.4 mg/kg QW and 0.8 mg/kg Q2W RP2Ds [14, 16]. Eligible patients were aged ≥ 18 years, had a documented diagnosis of RRMM, and had measurable disease per IMWG criteria [21]. In phase 1, patients had progressed on or were intolerant to all established therapies and had an Eastern Cooperative Oncology Group performance status (ECOG PS) of 0–1. In phase 2, patients had at least three prior LOTs, including an IMiD, PI, and anti‐CD38 antibody, and an ECOG PS of 0–2. Prior exposure to antibody–drug conjugates was permitted. Prior exposure to TCRT was not allowed in the QW and Q2W cohorts but was permitted in the separate prior TCRT cohort (treated with either the QW or Q2W schedule).

Exploratory analyses included CRS incidence after prolonged dosing intervals of talquetamab and characterization of immune phenotype. The interval analysis included patients from phases 1 and 2 of MonumenTAL‐1 who received talquetamab 0.4 mg/kg QW, Q2W, and monthly (Q4W) (*n* = 213) and 0.8 mg/kg QW, Q2W, and Q4W (*n* = 217). Immune phenotype was assessed in peripheral serum (cytokines) and whole blood (T‐cell markers); samples were collected prior to the first step‐up and after selected full treatment doses. T‐cell markers were assessed by flow cytometry; cytokines were measured by ELISA or MSD. Maximum fold change of cytokine induction and T‐cell activation was defined as the maximum increase fold change per patient observed at any time (up to cycle [C]2 day [D]1) compared with baseline. Statistical analyses were performed using the Wilcoxon rank‐sum test.

### 2.2 CRS Management

**FIGURE 1:**
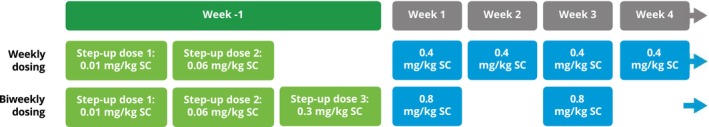
Dosing schedules at the RP2Ds in MonumenTAL‐1. RP2D, recommended phase 2 dose; SC, subcutaneous.

To reduce the risk of CRS, patients received step‐up doses of talquetamab at 0.01 and 0.06 mg/kg (QW) or 0.01, 0.06, and 0.3 mg/kg (Q2W) (Figure 1) separated by 2–4 days and completed 2–4 days prior to the first full treatment dose. Pretreatment with a glucocorticoid (dexamethasone 16 mg or equivalent), antihistamine (diphenhydramine 50 mg or equivalent), and antipyretic (acetaminophen 650–1000 mg or equivalent) was required prior to step‐up and initial full doses. Patients were required to be hospitalized for ≥ 48 h from the start of step‐up and first full doses and closely monitored for CRS or systemic administration‐related reactions. Management guidelines for CRS were provided in the study protocol (Table S1). Before talquetamab administration, CRS (including fever) had to have fully resolved, and patients must have had no evidence of serious bacterial, viral, or fungal infections.

CRS reported in phase 1 was graded according to the CRS revised grading system [22]. CRS reported in phase 2 was graded by the investigator according to ASTCT guidelines. For this analysis, the CRS revised grading system criteria were mapped to ASTCT criteria [3].

### 2.3 Statistical Analysis

Statistical methods have been described previously [14, 16]. Descriptive statistics were used for the incidence of CRS and analyses by grade, timing, and use of supportive measures.

## 3 Results

### 3.1 Patients

As of January 17, 2023, 288 patients with no prior TCRT received talquetamab 0.4 mg/kg QW (*n* = 143; median follow‐up, 18.8 months) or 0.8 mg/kg Q2W (*n* = 145; median follow‐up, 12.7 months). Fifty‐one patients in the prior TCRT cohort (median follow‐up, 14.8 months) received either QW (*n* = 43) or Q2W (*n* = 8). Of the 51 patients, 36 received a CAR‐T therapy and 18 received a bsAb (94% B‐cell maturation antigen [BCMA]–targeted); three patients received both. Patient demographics and disease characteristics have been previously published [14, 16] and are summarized in Table S2.

Excluding patients who received additional step‐up doses (i.e., repeat step‐up dosing) before the first full dose in C1, the median (range) time from step‐up dose 1 to 2 was 2.8 (1–7) days and from step‐up dose 2 to C1D1 was 3.0 (1–8) days for the QW cohort; for the Q2W cohort, there were 2.0 (1–8), 2.9 (1–7), and 2.9 (1–12) days from step‐up dose 1 to 2, step‐up dose 2 to 3, and step‐up dose 3 to C1D1, respectively.

### 3.2 Incidence, Timing, and Severity of CRS

CRS incidence was similar across cohorts (74.5%–79.0%; Table 1). Common symptoms were pyrexia (73.8%–79.0%), hypotension (13.8%–21.6%), and chills (9.1%–17.6%). CRS events were primarily low‐grade, with few grade 3 (0.7%–2.1%) and no grade 4/5 events. The proportion of patients who experienced grade ≥ 2 CRS was the same in the QW and Q2W cohorts (17% each). The incidence of grade 1 CRS prior to grade ≥ 2 CRS was low and similar across the QW (5/24 [21%]) and Q2W (6/25 [24%]) cohorts. Similar results were observed in the prior TCRT cohort for patients with grade ≥ 2 events (12/51 [24%]) and those with grade 1 prior to grade ≥ 2 CRS (3/12 [25%]).

**TABLE 1:** Overall incidence and severity of CRS.

Parameter	Talquetamab 0.4 mg/kg SC QW (*n* = 143)	Talquetamab 0.8 mg/kg SC Q2W (*n* = 145)	Prior TCRT (*n* = 51)
Patients with CRS, *n* (%)	113 (79.0)	108 (74.5)	39 (76.5)
Maximum toxicity grade, *n* (%)			
Grade 1	89 (62.2)	83 (57.2)	27 (52.9)
Grade 2	21 (14.7)	24 (16.6)	11 (21.6)
Grade 3	3 (2.1)	1 (0.7)	1 (2.0)
Grade 4/5	0	0	0
Patients with serious CRS^a^, *n* (%)	24 (16.8)	15 (10.3)	6 (11.8)
CRS leading to discontinuation, *n* (%)	0	1 (0.7)	0
Time to onset (h)^b^, ^c^, median (range)	25.9 (1.3–165.0)	28.0 (0.1–333.4)	26.3 (4.9–97.2)
Duration (h)^c^, median (range)	14.5 (0.5–221.6)	18.0 (0.0–621.8)	20.4 (0.9–71.5)
Patients with CRS up to first full dose, *n* (%)			
First step‐up dose	48 (33.6)	38 (26.2)	12 (23.5)
Second step‐up dose	70 (49.0)	59 (40.7)	21 (41.2)
Third step‐up dose	N/A	50 (34.5)	1 (2.0)
First full dose	38 (26.6)	19 (13.1)	17 (33.3)
Patients with CRS during cycle ≥ 2, *n* (%)	5 (3.5)	5 (3.4)	2 (3.9)
Patients with CRS during repeat step‐up dosing^d^, *n* (%)	0	3 (10.7)	1 (20.0)
Patients with first CRS up to first full dose, *n* (%)			
First step‐up dose	48 (33.6)	38 (26.2)	12 (23.5)
Second step‐up dose	53 (37.1)	39 (26.9)	16 (31.4)
Third step‐up dose	N/A	22 (15.2)	0
First full dose	8 (5.6)	5 (3.4)	11 (21.6)
Patients with first CRS during cycle ≥ 2, *n* (%)	0	3 (2.1)	0
Repeat step‐up for first CRS	0	0	0
Average inpatient stay (days), *n* (range)	7.5 (2–36)	9.0 (2–20)	8.0 (2–19)
Patients who received supportive measures^e^, *n* (%)	106 (74.1)	103 (71.0)	39 (76.5)
Tocilizumab^f^	50 (35.0)	55 (37.9)	26 (51.0)
Corticosteroids	5 (3.5)	5 (3.4)	8 (15.7)
Oxygen^g^	8 (5.6)	10 (6.9)	3 (5.9)
Nasal cannula low‐flow (≤ 6 L/min)	8 (5.6)	9 (6.2)	2 (3.9)
Face mask	0	0	1 (2.0)
Venturi mask	1 (0.7)	0	0
Other	0	1 (0.7)	0
Vasopressor	2 (1.4)	1 (0.7)	1 (2.0)
Patients with > 1 CRS event, *n* (%)	46 (32.2)	46 (31.7)	13 (25.5)
Grade worsened at any subsequent event	6 (4.2)	6 (4.1)	3 (5.9)

Abbreviations: CRS, cytokine release syndrome; N/A, not applicable; Q2W, every other week; QW, weekly; SC, subcutaneous; TCRT, T‐cell redirection therapy.

^a^ Defined as inpatient hospitalization or prolongation of existing hospitalization (for > 24 h).

^b^ Relative to the most recent dose.

^c^ Calculated only in phase 2 patients (timing was not uniformly collected in phase 1); *n =* 141, 140, and 36, respectively, for time to onset, and *n =* 139, 138, and 36, respectively, for duration of CRS.

^d^ Calculated based on patients who received repeat step‐up dosing (*n* = 8, 28, and 5, respectively).

^e^ Patients could receive more than one supportive therapy.

^f^ Tocilizumab was allowed for all CRS events; the protocol prohibited prophylactic tocilizumab use.

^g^ Primarily low flow.

Of 36 patients in the prior TCRT cohort who received prior CAR‐T therapy, 75.0% experienced CRS (grade 1, 55.6%; grade 2, 16.7%; grade 3, 2.0%); of 18 patients who received prior bsAb therapy, 72.2% experienced CRS (grade 1, 44.4%; grade 2, 27.8%).

CRS occurred concurrently with infections in three (2.7%), nine (8.3%), and 0 patients in the QW, Q2W, and prior TCRT cohorts, respectively; CRS occurred concurrently with neutropenia in nine (8.0%), two (1.9%), and three (7.7%) patients, respectively.

At data cutoff, all CRS events had resolved, except in one patient in the QW cohort. This 75‐year‐old male patient had grade 3 CRS during step‐up dose 2 concurrently with an infection and died due to the infection before the CRS event resolved. One 78‐year‐old female patient in the Q2W cohort, who had grade 3 hepatitis secondary to grade 1 CRS, discontinued treatment due to CRS. The CRS event resolved after talquetamab withdrawal and treatment with prednisolone.

The median time to onset and duration of CRS were captured consistently only in phase 2 for the QW, Q2W, and prior TCRT cohorts (*n* = 141, 140, and 36, respectively, for time to onset, and *n* = 139, 138, and 36, respectively, for duration of CRS). While the range of CRS onset was 0.1–333 h across cohorts, 89%–92% of events occurred within 48 h of the most recent talquetamab dose. The median duration of CRS events was similar across cohorts (15–20 [range 0–622] h) (Table 1).

### 3.3 Incidence of CRS Over Time

Most first CRS events occurred following step‐up dose 1 (24%–34%), 2 (27%–37%), 3 (15%; Q2W schedule only), and first full dose (3%–22%; Table 1). In the Q2W cohort, three patients had a first CRS event at C1D1, all grade 2 (Tables 1 and 2). There were no grade 2 events beyond C1D1, and no grade 3 events beyond C1D8 (Table 2).

**TABLE 2:** Incidence of CRS by grade and timing.

Patients with CRS events, *n* (%)	Talquetamab 0.4 mg/kg SC QW (*n =* 143)	Talquetamab 0.8 mg/kg SC Q2W (*n =* 145)	Prior TCRT (*n =* 51)
Total patients with CRS	113 (79.0)	108 (74.5)	39 (76.5)
Grade 1 CRS			
Step‐up dose 1	41 (28.7)	33 (22.8)	9 (17.6)
Step‐up dose 2	59 (41.3)	48 (33.1)	15 (29.4)
Step‐up dose 3	N/A	45 (31.0)	0
Cycle 1 day 1	34 (23.8)	16 (11.0)	13 (25.5)
Cycle 1 day 8	5 (3.5)	N/A	1 (2.0)
Cycle 1 day 15	2 (1.4)	7 (4.8)	0
Cycle 1 day 22	6 (4.2)	N/A	0
Cycle ≥ 2	5 (3.5)	5 (3.4)	2 (3.9)
Repeat step‐up	0	3 (2.1)	1 (2.0)
Grade 2 CRS			
Step‐up dose 1	7 (4.9)	7 (4.8)	4 (7.8)
Step‐up dose 2	12 (8.4)	12 (8.3)	6 (11.8)
Step‐up dose 3	N/A	4 (2.8)	1 (2.0)
Cycle 1 day 1	5 (3.5)	3 (2.1)	3 (5.9)
Cycle 1 day 8	0	N/A	0
Cycle 1 day 15	0	0	0
Cycle 1 day 22	0	N/A	0
Cycle ≥ 2	0	0	0
Repeat step‐up	0	0	0
Grade 3 CRS			
Step‐up dose 1	0	0	0
Step‐up dose 2	1 (0.7)	0	0
Step‐up dose 3	N/A	1 (0.7)	0
Cycle 1 day 1	1 (0.7)	0	1 (2.0)
Cycle 1 day 8	1 (0.7)	N/A	0
Cycle 1 day 15	0	0	0
Cycle 1 day 22	0	N/A	0
Cycle ≥ 2	0	0	0
Repeat step‐up	0	0	0

*Note:* CRS events reported in phase 1 were graded according to the CRS revised grading system described by Lee 2014 [22]. CRS events reported in phase 2 were graded by the investigator according to ASTCT guidelines; for this analysis, the Lee 2014 criteria were mapped to ASTCT criteria [3].

Abbreviations: ASTCT, American Society for Transplantation and Cellular Therapy; CRS, cytokine release syndrome; N/A, not applicable; Q2W, every other week; QW, weekly; SC, subcutaneous; TCRT, T‐cell redirection therapy.

### 3.4 CRS During Repeat Step‐Up Dosing

Across the QW, Q2W, and prior TCRT cohorts, eight (5.6%), 28 (19.3%), and five (9.8%) patients received a repeat step‐up dose, respectively, which is recommended after a dose hold or delay. Most repeat step‐up doses occurred after the first full dose (majority at C5+ in QW, C3–5+ in Q2W, and C1–2 in prior TCRT cohorts); median time between last dose received and repeat step‐up dose was 40–61 days across cohorts. Among patients who received a repeat step‐up dose, four experienced CRS (three in the Q2W [10.7%] and one in the prior TCRT [20.0%] cohort) (Table 1), all grade 1; CRS occurred during initial step‐up doses in three patients (all grade 1).

### 3.5 CRS After Prolonged Dosing Intervals

Among 213 patients receiving 0.4 mg/kg QW, Q2W, or Q4W who restarted talquetamab after prolonged dosing intervals, six (2.8%) reported grade 1 CRS with ≤ 28 days dosing interval (talquetamab restarted at full dose) and 0 reported CRS with 29–56 days dosing interval (talquetamab restarted at step‐up dose 2). Among 217 patients receiving 0.8 mg/kg QW, Q2W, or Q4W, 17 (7.8%) reported grade 1 CRS with ≤ 63 days dosing interval (talquetamab restarted at full dose); beyond day 63, no patients restarted talquetamab.

At the 0.8 mg/kg dose of patients who restarted talquetamab at the full treatment dose, pharmacokinetics (PK) analyses were performed to determine the impact of serum concentration on CRS development. Among evaluable patients (*n* = 8), two developed grade 1 CRS and had talquetamab serum concentrations below the PK threshold recommended by the FDA [23]. Six patients did not develop CRS; of these, three had concentrations below and three had concentrations above the PK threshold. Data were not available for the 0.4 mg/kg dose. Based on these limited results, it is inconclusive whether a target threshold concentration is needed to avoid CRS at the restart of talquetamab at the full treatment dose following a prolonged dosing interval.

### 3.6 Recurrent CRS Events

Recurrent CRS events occurred in 32.2%, 31.7%, and 25.5% of patients in the QW, Q2W, and prior TCRT cohorts, respectively (Table 1). Most occurred during step‐up dosing (11.9%, 15.0%, and 9.8%) and C1D1 (21.0%, 10.5%, and 11.8%), and fewer occurred beyond C1D1 (11.2%, 5.5%, and 5.9%). Median onset of recurrent CRS was 26–29 h from the last dose of talquetamab. Worsening of CRS at any subsequent event occurred in 4.2%, 4.1%, and 5.9% of patients in the QW, Q2W, and prior TCRT cohorts, respectively. In general, most patients who had a CRS event after C1D8 had experienced a previous CRS event with at least one step‐up or C1D1 dose; of these, most were grade 1, except one grade 3 event after C1D8, which occurred concurrently with bacteremia.

### 3.7 Management of CRS

Supportive measures for CRS were given to 71%–77% of patients and included tocilizumab, corticosteroids, oxygen, and vasopressors (Table 1).

Patients generally required one tocilizumab dose (Figure S1), and most CRS events were treated with tocilizumab without corticosteroids. A higher proportion of patients in the prior TCRT cohort received supportive measures, including tocilizumab with and without corticosteroids and corticosteroids without tocilizumab, compared with patients in the QW and Q2W cohorts (Table S3).

**FIGURE 2:**
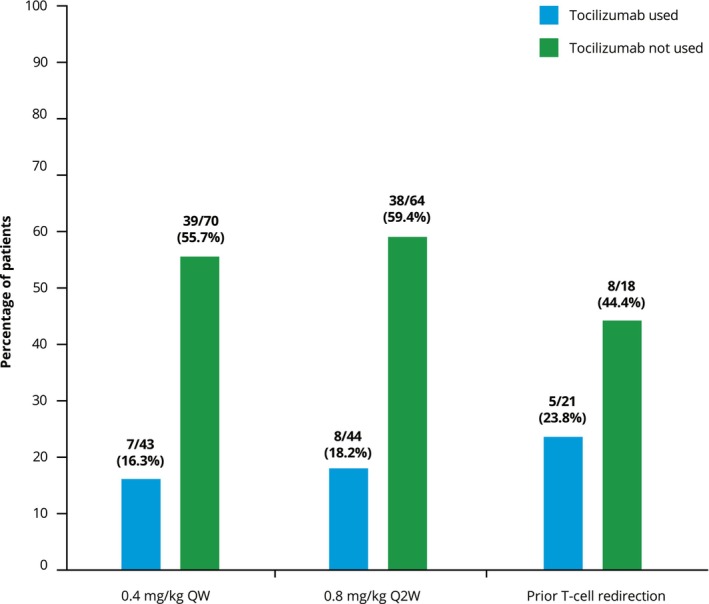
Proportion of patients experiencing subsequent CRS event(s) based on the use of tocilizumab to treat their first CRS event. CRS, cytokine release syndrome; Q2W, every 2 weeks; QW, weekly.

Across cohorts, 38%–54% of patients received tocilizumab for their first CRS event. The proportion of patients who experienced subsequent CRS events was 16%–24% for those who received tocilizumab for their first CRS event compared with 44%–59% for those who did not (Figure 2).

A higher proportion of patients who received tocilizumab for a CRS event before C2 experienced neutropenia (28%–69%) and infections (28%–54%) compared with patients who did not (8%–15% and 8%–16%, respectively). Interpretation of these data is limited by small numbers and the correlation of tocilizumab use with higher‐grade CRS, which is also a cause of neutropenia.

### 3.8 Impact on Efficacy

**FIGURE 3:**
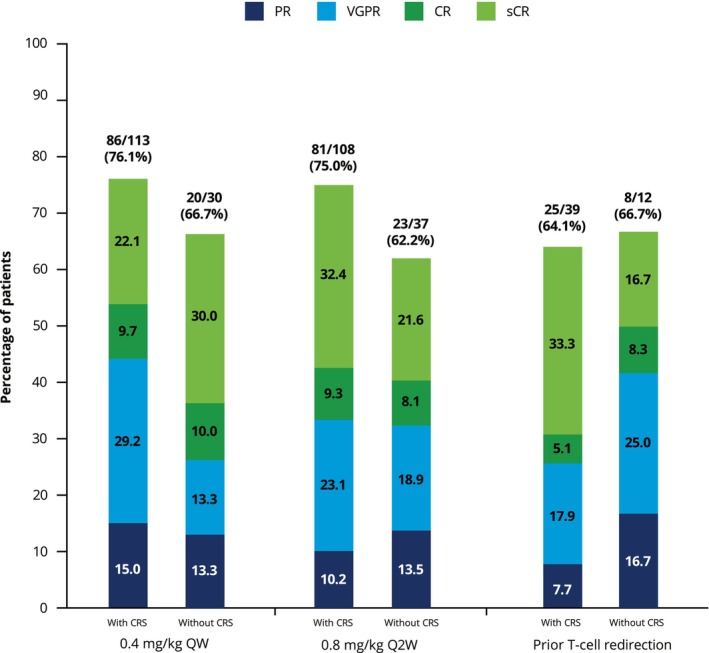
Overall best confirmed response in patients with and without CRS. CR, complete response; CRS, cytokine release syndrome; PR, partial response; Q2W, every 2 weeks; QW, weekly; sCR, stringent complete response; VGPR, very good partial response.

**FIGURE 4:**
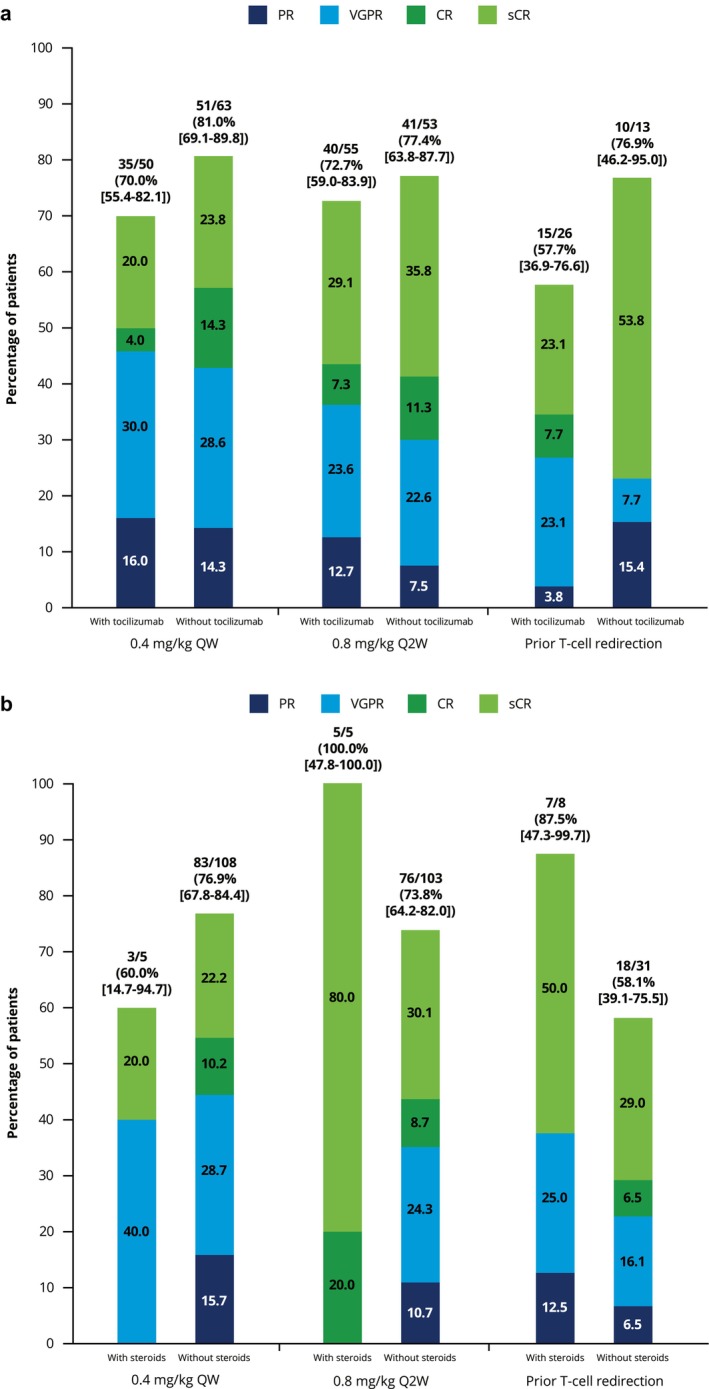
Impact of (a) tocilizumab and (b) corticosteroid treatment on ORR (% [95% CI]) in patients with CRS events. CI, confidence interval; CR, complete response; CRS, cytokine release syndrome; PR, partial response; Q2W, every 2 weeks; QW, weekly; sCR, stringent complete response; VGPR, very good partial response.

In the QW and Q2W cohorts, ORR was numerically higher in patients with versus without CRS, although the 95% CIs overlapped (Figure 3). Across cohorts, ORR was numerically higher in patients who did not receive tocilizumab treatment for CRS; again, the 95% CIs overlapped (Figure 4a). ORRs in patients who received corticosteroids ranged from 60.0% to 100.0% compared with 58.1% to 76.9% for those who did not receive corticosteroids (Figure 4b).

The median progression‐free survival (PFS) (95% CI) in patients who had a CRS event compared with those who did not was 7.6 (5.7–9.9) versus 7.5 (4.4 to not evaluable [NE]) months in the QW cohort; 14.2 (9.6–NE) versus 11.3 (2.5–NE) months in the Q2W cohort; and 5.0 (3.4–13.8) versus 5.1 (0.9–13.0) months in the prior TCRT cohort. The median PFS was numerically higher in patients who did not receive tocilizumab treatment for CRS compared with patients who did: 8.5 (6.7–12.1) versus 6.3 (4.9–7.7) months in the QW cohort; NE (14.2 months to NE) versus 11.9 (7.0–NE) months in the Q2W cohort; and 12.3 (3.8–NE) versus 3.9 (3.4–7.7) months in the prior TCRT cohort. Note that median PFS estimates were not mature in the Q2W and prior TCRT cohorts.

### 3.9 Baseline Characteristics and Immune Activation and CRS

Across the QW and Q2W cohorts, incidence, duration, recurrence, and severity of CRS events were similar and not impacted by baseline characteristics, including sex, race, International Staging System stage, presence of extramedullary disease, lymphocyte count, or percentage of bone marrow plasma cells. Results were generally consistent in the prior TCRT cohort, although patient subgroup numbers were smaller in this cohort (Table S4).

**FIGURE 5:**
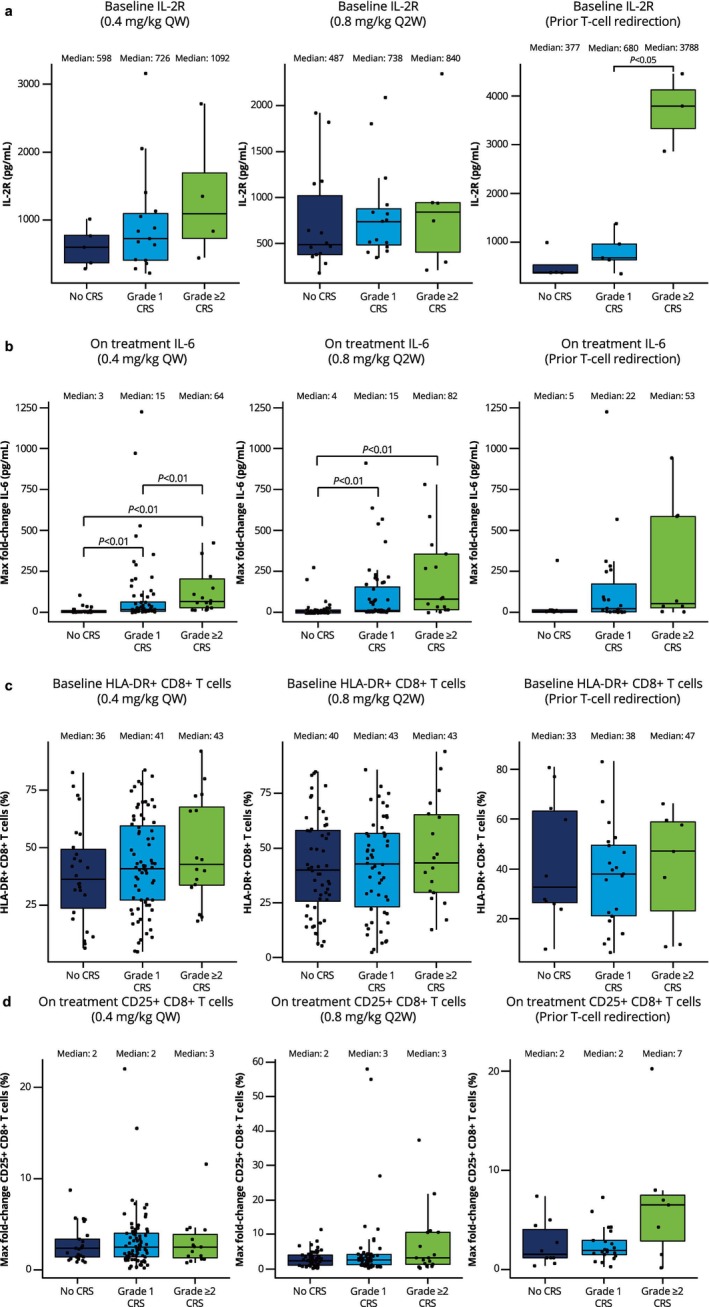
Correlations of cytokine levels and immune cell markers at baseline and on treatment (cycle 1) with CRS incidence and severity. (a) Baseline IL‐2R; (b) maximum fold change of IL‐6 on treatment; (c) baseline HLA‐DR+ CD8+ T cells; (d) maximum fold change of CD25+ CD8+ T cells on treatment. If *p* value is not depicted in figures, differences are not statistically significant. Maximum fold change was calculated from baseline to C2D1 (pre‐dose). For better visualization of the IL‐6 analysis, the *y*‐axis cut removed 3 points in the grade 1 CRS group and 2 points in the grade ≥ 2 CRS group (0.4 mg/kg QW) and 1 point in each group (0.8 mg/kg Q2W). CRS, cytokine release syndrome; HLA, human leukocyte antigen; IL‐2R, interleukin 2 receptor; IL‐6, interleukin 6; Q2W, every 2 weeks; QW, weekly.

In terms of cytokine profile, trends for higher baseline levels of IL‐2R expression were observed in patients with CRS and were most pronounced in patients with more severe (grade ≥ 2) CRS (Figure 5a). Grade ≥ 2 CRS was also associated with elevated baseline levels of IL‐10 and IFNα (QW and Q2W cohorts) and IFNγ (prior TCRT). On study (during C1), CRS was associated with higher induction of IL‐6 (significant in QW and Q2W cohorts, Figure 5b) and a trend for higher induction of IFNγ, TNFα, and soluble IL‐2Rα; again, higher induction was observed with grade ≥ 2 CRS.

In terms of immune markers, CRS was associated with lower baseline counts of peripheral CD8+ T cells (note data were limited in the prior TCRT cohort) and higher baseline proportions of CD8+ T cells expressing HLA‐DR (Figure 5c). In the QW and Q2W cohorts, CRS was also associated with higher baseline proportions of CD38+ regulatory T cells (Tregs) and CD4+ or CD8+ T cells expressing CD25, CD38, LAG‐3, TIM‐3, and PD‐1. On study, a trend for higher induction of CD25 (Figure 5d), LAG‐3, TIM‐3, and PD‐1 on T cells was associated with CRS incidence across cohorts. In both baseline and on‐study immune marker evaluations, trends were more pronounced with grade ≥ 2 CRS.

## 4 Discussion

In MonumenTAL‐1, CRS incidence (~75%), severity (mainly grade 1/2), onset (~1 day), and duration (~17 h) with talquetamab were consistent with other bsAb TCRT in RRMM, including teclistamab. Most CRS events occurred during step‐up and C1D1 doses. There were no grade 2 events beyond C1D1 and no grade 3 events beyond C1D8. Although rare, three patients in the Q2W cohort had a first CRS event at C1D1, all grade 2; physicians should thus be vigilant, as patients could still be at risk for CRS even if an event did not occur during step‐up dosing. Results in the prior TCRT cohort were similar to those in TCRT‐naïve patients, suggesting that any potential mechanisms of resistance developed against BCMA‐targeted TCRT did not impact the incidence or timing of CRS with talquetamab. Together, these data support the applicability of consensus statements for teclistamab to talquetamab, including the need for monitoring and early treatment of CRS during step‐up and immediately after C1D1 doses.

Talquetamab and teclistamab were both developed using the DuoBody platform and have the same CD3 arm, leading to similarities in CRS outcomes in the MonumenTAL‐1 and MajesTEC‐1 studies [7]. The incidence of CRS was 74.5%–79.0% in MonumenTAL‐1 and 72.1% in MajesTEC‐1. Discontinuations due to CRS were low (one with talquetamab and none with teclistamab) [7]. The median time to onset of CRS was 25.9–28.0 h with talquetamab and 31.1 h with teclistamab; the median duration was 14.5–20.4 h and 11.8 h, respectively [7]. A higher proportion of patients had the first CRS occurrence at C1D1 of talquetamab (3.4%–21.6%) and during or after C2 (2.1%) compared with teclistamab, where most patients had their first CRS occurrence at step‐up dose 1 (43.6%) or 2 (19.4%), and none had CRS after C2 [7]. These differences may be related to increased intervals between step‐up and first full treatment doses or distribution and levels of GPRC5D versus BCMA. Note that previous exposure‐response analyses in the MonumenTAL‐1 study showed no correlation of any grade or grade ≥ 2 CRS with talquetamab exposure; neither the probability of a patient experiencing CRS nor CRS severity was associated with peak talquetamab exposure at any step‐up or full treatment dose [24].

Recently, recommendations for reinitiating teclistamab following prolonged dosing intervals have been published, with no repeat step‐up dose recommended with ≤ 62 days dosing interval, and a repeat step‐up dose recommended for ≥ 63 days dosing interval [25]. A single‐center experience has shown that repeat step‐up dosing and inpatient monitoring after a median 35 days dose delay of both teclistamab and talquetamab may not be needed, as no CRS events among 33 patients were observed following restart of either therapy at full treatment dose [26]. In alignment with these data, our analysis showed minimal CRS development with restart of talquetamab at full dose following dosing intervals of ≤ 63 days, and overall lower risk of CRS when restarting versus initiating talquetamab for the first time. Supportive PK analyses did not identify a minimal target talquetamab serum concentration threshold needed to prevent CRS at restart, although sample size was small in the analysis.

Across talquetamab cohorts, patients who received tocilizumab for any grade CRS were less likely to experience subsequent CRS events than patients who did not. Recent studies have shown that tocilizumab prophylaxis can reduce CRS incidence and severity in patients with lymphomas, acute lymphoblastic leukemia, and prostate carcinoma who receive TCRT [27, 28, 29, 30]. In patients with RRMM, teclistamab, cevostamab, a bsAb targeting Fc receptor‐homolog 5 and CD3, and more recently, talquetamab, have shown that prophylactic tocilizumab may reduce the incidence of CRS without impacting antimyeloma activity [7, 31, 32, 33]. These data have important implications in terms of facilitating outpatient dosing of bsAbs including talquetamab, thereby reducing the burden of hospitalization and improving patient experience. IMWG guidelines include data supporting prophylactic tocilizumab use with bsAb in RRMM but currently do not endorse it beyond clinical trial investigations [12]. Larger randomized studies are needed to validate the results observed in the studies noted above. Overall, the data in the current report support the use of tocilizumab for the management of CRS following talquetamab as used in the MonumenTAL‐1 protocol.

Resolution of CRS with tocilizumab has been shown to occur without detrimental effect on efficacy (or safety), as demonstrated in patients treated with TCRT in RRMM and other patient populations [7, 30, 31, 34, 35, 36]. In the current study, patient numbers were relatively low and 95% CIs overlapped in the ORR analysis, indicating that ORRs were comparable irrespective of tocilizumab administration; PFS data were similar to the response data but again, patient numbers were low, and data were not mature in the Q2W and prior TCRT cohorts, making it difficult to draw definitive conclusions. In MajesTEC‐1, ORRs with teclistamab did not appear to be impacted by tocilizumab administration (70.0% with tocilizumab vs. 74.0% without) [7]; the impact of tocilizumab on PFS with teclistamab was not evaluated. Although these analyses were exploratory, tocilizumab administration likely does not impact response or durability of response to talquetamab.

Our analyses did not identify significant associations between baseline characteristics and CRS parameters. However, trends in association with immune correlatives were observed. In patients with vs. those without CRS, baseline analyses revealed lower CD8+ T‐cell counts and higher frequencies of Tregs and co‐inhibitory receptor expression on T cells; on‐treatment analyses (during C1) revealed higher induction of markers associated with T‐cell activation. These trends were more pronounced in patients with more severe CRS. Cytokine data showed trends for both higher baseline and higher induction of cytokines during C1 to correlate with CRS incidence and severity (not statistically significant). However, no one marker appeared to clearly predict for CRS occurrence or severity, and no notable differences were observed between TCRT‐naïve and TCRT‐exposed populations. Future efforts are needed to identify predictive markers of CRS to inform clinical practice.

Limitations to these analyses include the single‐arm design of MonumenTAL‐1; the duration of follow‐up was shorter in the Q2W than QW cohort; the analysis was exploratory with small patient numbers; there were few non‐White participants. Despite this, similar trends observed in the MajesTEC‐1 trial support these results [7] and suggest that, due to the class effect, management of CRS can be applied across the agents studied.

## 5 Conclusion

CRS outcomes with talquetamab were consistent with those with teclistamab, allowing clinicians to leverage similar strategies to manage CRS in patients with RRMM. Guidance on restarting talquetamab following prolonged dosing intervals was also provided. Together, these results support confidence in the use of talquetamab as the only GPRC5D bsAb approved in RRMM.

## Author Contributions

**Niels W. C. J. van de Donk:** conceptualization, investigation, data curation, writing – original draft, writing – review and editing. **Ajai Chari:** conceptualization, investigation, data curation, writing – original draft, writing – review and editing. **Thomas Martin:** conceptualization, investigation, data curation, writing – original draft, writing – review and editing. **Amrita Krishnan:** conceptualization; investigation; data curation; writing – original draft, writing – review and editing. **Leo Rasche:** conceptualization, investigation, data curation, writing – original draft, writing – review and editing. **Jing Christine Ye:** conceptualization, investigation, data curation, writing – original draft, writing – review and editing. **Rakesh Popat:** conceptualization, investigation, data curation, writing – original draft, writing – review and editing. **Brea Lipe:** conceptualization, investigation, data curation, writing – original draft, writing – review and editing. **Cesar Rodriguez:** conceptualization, investigation, data curation, writing – original draft, writing – review and editing. **Carolina Schinke:** conceptualization, investigation, data curation, writing – original draft, writing – review and editing. **Sheri Skerget:** methodology, data curation, formal analysis, visualization, writing – review and editing. **Deeksha Vishwamitra:** methodology, data curation, formal analysis, visualization, writing – review and editing. **Raluca Verona:** methodology, investigation, writing – review and editing. **Jue Gong:** methodology, data curation, formal analysis, writing – review and editing. **Indrajeet Singh:** methodology, data curation, formal analysis, writing – review and editing. **Michela Campagna:** methodology, investigation, writing – review and editing. **Tara Masterson:** methodology, investigation, writing – review and editing. **Brandi Hilder:** methodology, investigation, writing – review and editing. **Jaszianne Tolbert:** methodology, investigation, writing – review and editing. **Thomas Renaud:** methodology, investigation, writing – review and editing. **M. Damiette Smit:** methodology, investigation, writing – review and editing. **Christoph Heuck:** methodology, investigation, writing – review and editing. **Maria‐Victoria Mateos:** conceptualization, investigation, data curation, writing – original draft, writing – review and editing.

## Ethics Statement

The study was conducted in accordance with the principles of the Declaration of Helsinki and the Good Clinical Guidelines of the International Council for Harmonisation. The protocol and amendments were approved by the institutional review board at each study site, as shown in the table below:

**Table:** 

Country	Ethics Committee/Institutional Review Board Site	Approval date	Site ID Ref #
Belgium	J43‐BE10003	20 Apr 2021	3680010
Belgium	U77‐BE10004	07 Apr 2021	3680010
Belgium	U77‐BE10002	23 Apr 2021	3680010
Belgium	U77‐BE10001	30 Apr 2021	3680010
Belgium	U77‐BE10003	07 Apr 2021	3680010
China	U77‐CN10005	24 Jan 2022	60868387
China	U77‐CN10007	16 Mar 2022	151734838/42909810
China	U77‐CN10008	19 Dec 2022	4578301
China	U77‐CN10002	21 Feb 2022	67081757
China	U77‐CN10011	28 Jun 2023	296960146
China	U77‐CN10001	07 Jan 2022	60868406
China	U77‐CN10003	04 Mar 2022	107910421
China	U77‐CN10004	25 Jan 2022	60890218/60868387
France	U77‐FR10001	23 Feb 2021	109642093
France	U77‐FR10002	15 Apr 2021	109642093
France	U77‐FR10003	20 Apr 2021	109642093
France	U77‐FR10006	19 Apr 2021	109642093
France	U77‐FR10004	23 Feb 2021	109642093
France	U77‐FR10005	14 Apr 2021	109642093
Germany	U77‐DE10001	10 May 2021	3619686
Germany	U77‐DE10005	04 Jun 2021	143018264/3619686
Germany	U77‐DE10003	20 Apr 2021	65501442/3619686
Germany	U77‐DE10002	11 Jun 2021	1966647/3619686
Israel	U77‐IL10003	30 Mar 2021	67104354
Israel	U77‐IL10001	16 Mar 2021	65304579
Israel	U77‐IL10002	17 Mar 2021	214624638
Israel	U77‐IL10004	11 Apr 2021	65304587
Israel	U77‐IL10005	20 Apr 2021	65304587
Japan	U77‐JP10008	04 Jul 2022	24221094
Japan	U77‐JP10004	19 Jul 2022	61088794
Japan	U77‐JP10012	20 Sep 2022	233581552
Japan	U77‐JP10005	05 Sep 2022	4607595
Japan	U77‐JP10011	20 Jul 2022	50981609
Japan	U77‐JP10013	08 Nov 2022	112622177
Japan	U77‐JP10009	11 Jul 2022	61088673
Japan	U77‐JP10003	14 Jun 2022	200315467
Japan	U77‐JP10010	22 Sep 2022	4607379
Japan	U77‐JP10014	23 Aug 2023	290626781
Japan	U77‐JP10006	31 Jul 2022	2454817
Japan	U77‐JP10015	12 Sep 2023	162750144
Japan	U77‐JP10007	19 Sep 2022	214786450
Netherlands	J43‐NL10001	05 Sep 2019	67390905/61279424
Netherlands	J43‐NL10002	11 Feb 2019	61279424
Netherlands	U77‐NL10001	08 Jan 2021	2671572
Netherlands	U77‐NL10002	29 Jan 2021	2671572
Poland	U77‐PL10003	12 Apr 2021	295430681
Poland	U77‐PL10001	13 Apr 2021	295430681
Poland	U77‐PL10004	13 Apr 2021	295430681
Poland	U77‐PL10005	13 Apr 2021	295430681
Poland	U77‐PL10002	13 Apr 2021	295430681
Republic of Korea	U77‐KR10003	07 Apr 2021	87655320
Republic of Korea	U77‐KR10006	07 Apr 2021	9933063
Republic of Korea	U77‐KR10002	07 Apr 2021	3267750
Republic of Korea	U77‐KR10004	07 Apr 2021	3763615
Republic of Korea	U77‐KR10001	07 Apr 2021	3268760
Republic of Korea	U77‐KR10005	17 Apr 2021	3267957
Spain	J43‐ES10001	24 May 2018	7830447
Spain	J43‐ES10006	23 Apr 2021	7830447
Spain	J43‐ES10004	23 Jul 2019	7830447
Spain	J43‐ES10002	28 Feb 2019	7830447
Spain	J43‐ES10003	26 Apr 2018	7830447
Spain	U77‐ES10006	02 Mar 2021	102343062
Spain	U77‐ES10002	08 Feb 2021	102343062
Spain	U77‐ES10003	17 Feb 2021	102343062
Spain	U77‐ES10009	18 Mar 2021	102343062
Spain	U77‐ES10001	03 Mar 2021	102343062
Spain	U77‐ES10010	11 Feb 2021	102343062
Spain	U77‐ES10005	04 Jun 2021	102343062
Spain	U77‐ES10011	19 Feb 2021	102343062
Spain	U77‐ES10004	16 Feb 2021	102343062
Spain	U77‐ES10007	10 Mar 2021	102343062
Spain	U77‐ES10008	28 Apr 2021	102343062
US	J43‐US10005	11 Jul 2018	75157813
US	J43‐US10009	02 Mar 2018	117606562
US	J43‐US10003	16 Dec 2017	18210329
US	J43‐US10007	28 Oct 2020	246139841
US	J43‐10001	05 Feb 2018	246139841
US	U77‐US10011	15 Apr 2021	246139841
US	U77‐US10015	27 Apr 2021	72485809
US	U77‐US10005	23 Jul 2021	4475249
US	U77‐US10026	31 Mar 2021	3615478
US	U77‐US10013	18 Feb 2021	3615478
US	U77‐US10016	09 Jun 2021	2630545
US	U77‐US10022	28 Jun 2021	3615478
US	U77‐US10024	01 Apr 2021	28196129
US	U77‐US10007	14 Apr 2021	3615478
US	U77‐US10014	13 May 2021	4783017
US	U77‐US10009	14 Jan 2021	61089064/117606562
US	U77‐US10003	28 Jan 2021	60967175
US	U77‐US10025	07 May 2021	3615478

## Consent

All patients provided written informed consent for this study.

## Conflicts of Interest

Niels W. C. J. van de Donk has received research funding from Amgen, BMS, Celgene, Cellectis, Johnson & Johnson, and Novartis; and reports a consulting or advisory role with AbbVie, Adaptive Biotechnologies, Amgen, Bayer, BMS, Celgene, Galapagos, Johnson & Johnson, Novartis, Pfizer, Roche, Servier, and Takeda. Ajai Chari is a consultant for Amgen, Antengene, BMS, Johnson & Johnson, Millenium/Takeda, and Secura Bio; is a member of an advisory board for AbbVie, Amgen, BMS, Genentech, GSK, Johnson & Johnson, Karyopharm, Sanofi, Seattle Genetics, Secura Bio, and Shattuck Labs; and has received research funding from Amgen, BMS, Johnson & Johnson, Millenium/Takeda, and Seattle Genetics. Thomas G. Martin has served as a consultant or in an advisory role for GSK and Legend Biotech; and has received research funding from Sanofi, Amgen, and Johnson & Johnson. Amrita Y. Krishnan received research funding from Johnson & Johnson; holds equity in BMS; served as a consultant for Adaptive, Johnson & Johnson, Sanofi, BMS, Pfizer, and Regeneron; served on a speakers bureau for Takeda, GSK, and BMS; and served on an advisory committee for Sutro. Leo Rasche has received honoraria from BMS, GSK, Johnson & Johnson, Pfizer, Roche, and Sanofi; is a member of a board of directors or advisory committee for Amgen, BMS, Johnson & Johnson, Pfizer, and Sanofi; is a consultant for Amgen, BMS, GSK, Johnson & Johnson, Pfizer, and Sanofi; and has received research funding from BMS and Skyline Dx. Jing Christine Ye has served in a consulting or advisory role for BMS and Johnson & Johnson; has received honoraria from BMS and Johnson & Johnson; and has received research funding from Celgene, Genmab, GSK, MingSight, Novartis, Pfizer, and Regeneron. Rakesh Popat has served in a consulting or advisory role for GSK, Celgene, Roche, BeiGene, and Johnson & Johnson; has received travel, accommodations, and expenses from Johnson & Johnson and GSK; received honoraria from Johnson & Johnson, Celgene, GSK, AbbVie, and Sanofi; and has received research funding from GSK. Brea Lipe reports a consulting or advisory role with BMS, GSK, Johnson & Johnson, Karyopharm Therapeutics, Sanofi, and Takeda; and has received research funding from Amgen, Celgene, Cellectar, Johnson & Johnson, Karyopharm Therapeutics, and Seagen. Cesar Rodriguez reports personal fees from Amgen, BMS, and Takeda. Carolina Schinke has no conflicts of interest to disclose. Deeksha Vishwamitra is an employee of Johnson & Johnson and reports stock and other ownership interests in Johnson & Johnson. Sheri Skerget, Raluca Verona, Jue Gong, Indrajeet Singh, Michela Campagna, Tara Masterson, Brandi W. Hilder, Jaszianne Tolbert, Thomas Renaud, and Christoph Heuck are employees of Johnson & Johnson and may hold stock, stock options, or equity in Johnson & Johnson. M. Damiette Smit was an employee of Johnson & Johnson at the time this work was performed; is a current employee of Enliven Therapeutics; and reports patents at Johnson & Johnson and Amgen. María‐Victoria Mateos has received honoraria from AbbVie, Amgen, BMS/Celgene, GSK, Johnson & Johnson, Oncopeptides, Pfizer, Regeneron, Sanofi, Stemline, and Takeda; is a member of a board of directors or advisory committee for AbbVie, BMS/Celgene, GSK, Johnson & Johnson, Oncopeptides, Pfizer, Sanofi, Stemline, and Takeda; and is a speakers' bureau member for Johnson & Johnson.

## Supporting information


**Table S1:** Guidelines for the management of CRS as defined in the MonumenTAL‐1 clinical protocol.
**Table S2:** Patient demographics and disease characteristics.
**Table S3:** Patients receiving tocilizumab and/or corticosteroids for treatment‐emergent CRS.
**Table S4:** CRS by baseline patient demographics and disease characteristics.
**Figure S1:** Percentage of patients who required one, two, or three doses of tocilizumab.

## Data Availability

The data sharing policy of Johnson & Johnson is available at https://innovativemedicine.jnj.com/our‐innovation/clinical‐trials/transparency. As noted on this site, requests for access to the study data can be submitted through Yale Open Data Access (YODA) Project site at http://yoda.yale.edu.
