# Supplementary material for: Characterization and Management of Cytokine Release Syndrome From the MonumenTAL‐1 Study of Talquetamab in Patients With Relapsed/Refractory Multiple Myeloma
Source: Cancer Med. 2025 Oct 2;14(19):e71276. doi: 10.1002/cam4.71276 (PMC12489744; doi:10.1002/cam4.71276)
Supplement: Supplementary file 1 — Table S1: Guidelines for the management of CRS as defined in the MonumenTAL‐1 clinical protocol. Table S2: Patient demographics and disease characteristics. Table S3: Patients receiving tocilizumab and/or corticosteroids for treatment‐emergent CRS. Table S4: CRS by baseline patient demographics and disease characteristics. Figure S1: Percentage of patients who required one, two, or three doses of tocilizumab. [file CAM4-14-e71276-s001.docx]

Characterization and management of cytokine release syndrome from the MonumenTAL-1 study of talquetamab in patients with relapsed/refractory multiple myeloma

# SUPPLEMENTARY APPENDIX

## SUPPLEMENTARY TABLE S1 Guidelines for the management of CRS as defined in the MonumenTAL-1 clinical protocol.

| **Presenting symptoms** | **Treatment options** | |
| --- | --- | --- |
|  | **Tocilizumab** | **Corticosteroids** |
| Temperature ≥38 °C^a^ | May be considered | N/A |
| Temperature ≥38 °C^a^ with either hypotension responsive to fluids and not requiring vasopressors or oxygen requirement of low-flow nasal cannula^b^ or blow-by | Administer tocilizumab 8 mg/kg IV over 1 hour (not to exceed 800 mg). Repeat tocilizumab every 8 hours as needed if not responsive to IV fluids or increasing supplemental oxygen. Limit to a maximum of three doses in a 24-hour period; maximum total of four doses | Manage per guidance below if no improvement within 24 hours of starting tocilizumab |
| Temperature ≥38 °C^a^ with either hypotension requiring one vasopressor with or without vasopressin or oxygen requirement of high-flow nasal cannula,^b^ facemask, or non-rebreather | Administer tocilizumab 8 mg/kg IV over 1 hour (not to exceed 800 mg). Repeat tocilizumab every 8 hours as needed if not responsive to IV fluids or increasing supplemental oxygen. Limit to a maximum of three doses in a 24-hour period; maximum total of four doses | If no improvement, administer methylprednisolone 1 mg/kg IV twice daily or equivalent dexamethasone (e.g., 10 mg IV every 6 hours). Continue corticosteroids use until the event is grade ≤1, then taper over 3 days |
| Temperature ≥38 °C^a^ with either hypotension requiring multiple vasopressors (excluding vasopressin) or oxygen requirement of positive pressure (e.g., CPAP, BiPAP, intubation, or mechanical ventilation) | Administer tocilizumab 8 mg/kg IV over 1 hour (not to exceed 800 mg). Repeat tocilizumab every 8 hours as needed if not responsive to IV fluids or increasing supplemental oxygen. Limit to a maximum of three doses in a 24-hour period; maximum total of four doses | As above or administer methylprednisolone 1000 mg IV per day for 3 days per investigator discretion. If no improvement or if condition worsens, consider alternative immunosuppressants^c^ |

Abbreviations: BiPAP, bilevel positive airway pressure; CPAP, continuous positive airway pressure; CRS, cytokine release syndrome; IV, intravenous; N/A, not applicable.

^a^Attributed to CRS. Fever may not always be present concurrently with hypotension or hypoxia as it may be masked by interventions such as antipyretics or anticytokine therapy (e.g., tocilizumab or steroids).

^b^Low-flow nasal cannula is ≤6 L/min, and high-flow nasal cannula is >6 L/min.

^c^Monoclonal antibodies targeting cytokines may be considered based on institutional practice for unresponsive CRS.

## SUPPLEMENTARY TABLE S2 Patient demographics and disease characteristics.

| **Characteristic** | **Talquetamab 0.4 mg/kg SC QW^a^ (*n =* 143)** | | **Talquetamab 0.8 mg/kg SC Q2W^a^ (*n =* 145)** | **Prior TCRT^a^  (*n =* 51)** |
| --- | --- | --- | --- | --- |
| Age (years), median (range) | 67.0 (46–86) | | 67.0 (38–84) | 61.0 (38–78) |
| Male, *n* (%) | 78 (54.5) | | 83 (57.2) | 31 (60.8) |
| Race, *n* (%) | |  | | |
| White | 128 (89.5) | | 125 (86.2) | 47 (92.2) |
| Black | 12 (8.4) | | 9 (6.2) | 3 (5.9) |
| Asian | 1 (0.7) | | 6 (4.1) | 1 (2.0) |
| Not reported | 2 (1.4) | | 2 (1.4) | 0 |
| Bone marrow plasma cells ≥60%,^b^ *n* (%) | 17 (12.3) | | 32 (22.7) | 8 (17.0) |
| Extramedullary plasmacytomas ≥1,^c^ *n* (%) | 33 (23.1) | | 37 (25.5) | 16 (31.4) |
| High-risk cytogenetics,^d^ *n* (%) | 41 (31.1) | | 37 (28.9) | 18 (40.9) |
| ISS stage, *n* (%)^e^ | |  | | |
| I | 62 (43.4) | | 64 (44.4) | 24 (47.1) |
| II | 53 (37.1) | | 45 (31.3) | 18 (35.3) |
| III | 28 (19.6) | | 35 (24.3) | 9 (17.6) |
| Time since diagnosis (years), median (range) | 6.7 (1.4–20.8) | | 6.4 (0.8–25.4) | 6.3 (1.7–19.6) |
| Prior LOTs, median (range) | 5 (2–13) | | 5 (2–17) | 6 (3–15) |
| Prior stem cell transplantation, *n* (%) | 113 (79.0) | | 114 (78.6) | 45 (88.2) |
| Exposure status, *n* (%) | |  | | |
| Triple-class^f^ | 143 (100.0) | | 145 (100.0) | 51 (100.0) |
| Penta-drug^g^ | 105 (73.4) | | 101 (69.7) | 40 (78.4) |
| Belantamab mafadotin | 22 (15.4) | | 16 (11.0) | 6 (11.8) |
| Bispecific antibody | N/A | | N/A | 18 (35.3) |
| CAR-T therapy | N/A | | N/A | 36 (70.6) |
| Refractory status, *n* (%) | |  | | |
| PI^h^ | 114 (79.7) | | 120 (82.8) | 46 (90.2) |
| IMiD^i^ | 133 (93.0) | | 130 (89.7) | 49 (96.1) |
| Anti-CD38 mAb^j^ | 133 (93.0) | | 134 (92.4) | 49 (96.1) |
| Triple-class^f^ | 106 (74.1) | | 100 (69.0) | 43 (84.3) |
| Penta-drug^g^ | 42 (29.4) | | 34 (23.4) | 21 (41.2) |
| Belantamab mafadotin | 18 (12.6) | | 13 (9.0) | 4 (7.8) |
| To last LOT | 134 (93.7) | | 137 (94.5) | 31 (60.8) |

Abbreviations: CAR-T, chimeric antigen receptor T cell; IMiD, immunomodulatory drug; ISS, International Staging System; LOT, line of therapy; mAb, monoclonal antibody; N/A, not applicable; PI, proteasome inhibitor; Q2W, every other week; QW, weekly; SC, subcutaneous; TCRT, T-cell redirection therapy.

^a^With two to three step-up doses.

^b^Percentages calculated from *n =* 138 for 0.4 mg/kg QW, *n =* 141 for 0.8 mg/kg Q2W, and
*n =* 47 for patients with prior TCR.

^c^Soft tissue plasmacytomas not associated with the bone were included.

^d^del(17p), t(4:14), and/or t(14;16); calculated from *n =* 132 for 0.4 mg/kg SC QW, *n =* 128 for 0.8 mg/kg SC Q2W, and *n =* 44 for patients with prior T-cell redirection therapies.

^e^Percentages calculated from *n =* 144 for 0.8 mg/kg Q2W.

^f^At least one PI, at least one IMiD, and at least one anti-CD38 mAb.

^g^Two or more PIs, two or more IMiDs, and at least one anti-CD38 mAb.

^h^Bortezomib, carfilzomib, and/or ixazomib.

^i^Thalidomide, lenalidomide, and/or pomalidomide.

^j^Daratumumab, isatuximab, and/or an investigational anti-CD38 mAb.

**SUPPLEMENTARY TABLE S3** Patients receiving tocilizumab and/or corticosteroids for treatment-emergent CRS.

| **Event, *n* (%)** | **Talquetamab 0.4 mg/kg SC QW^a^ (*n =* 143)** | **Talquetamab 0.8 mg/kg SC Q2W^a^ (*n =* 145)** | **Prior TCRT^a^ (*n =* 51)** |
| --- | --- | --- | --- |
| Number of patients with CRS | 113 (79.0) | 108 (74.5) | 39 (76.5) |
| Received tocilizumab but no corticosteroids^a^ | 48 (33.6) | 50 (34.5) | 23 (45.1) |
| Received corticosteroids but no tocilizumab^a^ | 2 (1.4) | 2 (1.4) | 5 (9.8) |
| Received both tocilizumab and corticosteroids^a^ | 3 (2.1) | 5 (3.4) | 4 (7.8) |
| Number of CRS events | 189 | 189 | 57 |
| Events treated with tocilizumab but   no corticosteroids | 56 (29.6) | 60 (31.7) | 27 (47.4) |
| Grade 1 | 40 (21.2) | 46 (24.3) | 20 (35.1) |
| Grade 2 | 15 (7.9) | 14 (7.4) | 7 (12.3) |
| Grade 3 | 1 (0.5) | 0 | 0 |
| Events treated with corticosteroids   but no tocilizumab | 2 (1.1) | 2 (1.1) | 6 (10.5) |
| Grade 1 | 2 (1.1) | 2 (1.1) | 2 (3.5) |
| Grade 2 | 0 | 0 | 4 (7.0) |
| Events treated with both   tocilizumab and corticosteroids | 5 (2.6) | 6 (3.2) | 4 (7.0) |
| Grade 1 | 2 (1.1) | 3 (1.6) | 1 (1.8) |
| Grade 2 | 2 (1.1) | 2 (1.1) | 2 (3.5) |
| Grade 3 | 1 (0.5) | 1 (0.5) | 1 (1.8) |

Abbreviations: CRS, cytokine release syndrome; QW, weekly; Q2W, every other week; SC, subcutaneous; TCRT, T-cell redirection therapy.

^a^For at least one event of CRS.

## SUPPLEMENTARY TABLE S4 CRS by baseline patient demographics and disease characteristics.^a^

|  | **Talquetamab 0.4 mg/kg SC QW  (*n =* 143)** | | | | | | **Talquetamab 0.8 mg/kg SC Q2W  (*n =* 145)** | | | | | | **Prior TCRT  (*n =* 51)** | | | | | |
| --- | --- | --- | --- | --- | --- | --- | --- | --- | --- | --- | --- | --- | --- | --- | --- | --- | --- | --- |
| **Sex** | **Male (*n =* 78)** | | | **Female (*n =* 65)** | | | **Male (*n =* 83)** | | | **Female (*n =* 62)** | | | **Male (*n =* 31)** | | | **Female (*n =* 20)** | | |
| Patients with CRS | 61 (78.2) | | | 52 (80.0) | | | 62 (74.7) | | | 46 (74.2) | | | 27 (87.1) | | | 12 (60.0) | | |
| Grade 1 | 48 (61.5) | | | 41 (63.1) | | | 46 (55.4) | | | 37 (59.7) | | | 20 (64.5) | | | 7 (35.0) | | |
| Grade 2 | 11 (14.1) | | | 10 (15.4) | | | 16 (19.3) | | | 8 (12.9) | | | 6 (19.4) | | | 5 (25.0) | | |
| Grade 3 | 2 (2.6) | | | 1 (1.5) | | | 0 | | | 1 (1.6) | | | 1 (3.2) | | | 0 | | |
| Grade ≥4 | 0 | | | 0 | | | 0 | | | 0 | | | 0 | | | 0 | | |
| Multiple CRS events | 21 (26.9) | | | 25 (38.5) | | | 26 (31.3) | | | 20 (32.3) | | | 7 (22.6) | | | 6 (30.0) | | |
| Median duration of CRS (days), *n* (range) | 2.0 (1-11) | | | 2.0 (1-13) | | | 2.0 (1-27) | | | 2.0 (1-29) | | | 2.0 (1-6) | | | 2.0 (1-4) | | |
| **Race** | **White  (*n =* 128)** | **Black  (*n =* 12)** | | **Asian  (*n =* 1)** | | **NR  (*n =* 2)** | **White  (*n =* 125)** | **Black  (*n =* 9)** | | **Asian  (*n =* 6)** | | **NR  (*n =* 5)** | **White  (*n =* 47)** | **Black  (*n =* 3)** | | **Asian  (*n =* 1)** | | **NR  (*n =* 0)** |
| Patients with CRS | 101 (78.9) | 9 (75.0) | | 1 (100.0) | | 2 (100.0) | 91 (72.8) | 6 (66.7) | | 6 (100.0) | | 5 (100.0) | 36 (76.6) | 3 (100.0) | | 0 | | 0 |
| Grade 1 | 79 (61.7) | 7 (58.3) | | 1 (100.0) | | 2 (100.0) | 69 (55.2) | 4 (44.4) | | 6 (100.0) | | 4 (80.0) | 24 (51.1) | 3 (100.0) | | 0 | | 0 |
| Grade 2 | 19 (14.8) | 2 (16.7) | | 0 | | 0 | 21 (16.8) | 2 (22.2) | | 0 | | 1 (20.0) | 11 (23.4) | 0 | | 0 | | 0 |
| Grade 3 | 3 (2.3) | 0 | | 0 | | 0 | 1 (0.8) | 0 | | 0 | | 0 | 1 (2.1) | 0 | | 0 | | 0 |
| Grade ≥4 | 0 | 0 | | 0 | | 0 | 0 | 0 | | 0 | | 0 | 0 | 0 | | 0 | | 0 |
| Multiple CRS events | 45 (35.2) | 0 | | 1 (100.0) | | 0 | 37 (29.6) | 3 (33.3) | | 3 (50.0) | | 3 (60.0) | 12 (25.5) | 1 (33.3) | | 0 | | 0 |
| Median duration of CRS (days), *n* (range) | 2.0  (1–13) | 2.0  (1–4) | | 2.5  (1–4) | | 3.0  (2–4) | 2.0  (1–29) | 2.0  (1–3) | | 2.0  (1–8) | | 2.0  (1–3) | 2.0  (1–6) | 2.0  (2–3) | | – | | – |
| **Cytogenetics** | **High-risk (*n =* 41)** | | | **Standard-risk  (*n =* 91)** | | | **High-risk (*n =* 37)** | | | **Standard-risk  (*n =* 91)** | | | **High-risk (*n =* 18)** | | | **Standard-risk  (*n =* 26)** | | |
| Patients with CRS | 32 (78.0) | | | 73 (80.2) | | | 28 (75.7) | | | 67 (73.6) | | | 16 (88.9) | | | 16 (61.5) | | |
| Grade 1 | 25 (61.0) | | | 57 (62.6) | | | 20 (54.1) | | | 52 (57.1) | | | 13 (72.2) | | | 11 (42.3) | | |
| Grade 2 | 7 (17.1) | | | 14 (15.4) | | | 7 (18.9) | | | 15 (16.5) | | | 2 (11.1) | | | 5 (19.2) | | |
| Grade 3 | 0 | | | 2 (2.2) | | | 1 (2.7) | | | 0 | | | 1 (5.6) | | | 0 | | |
| Grade ≥4 | 0 | | | 0 | | | 0 | | | 0 | | | 0 | | | 0 | | |
| Multiple CRS events | 15 (36.6) | | | 27 (29.7) | | | 14 (37.8) | | | 26 (28.6) | | | 7 (38.9) | | | 3 (11.5) | | |
| Median duration of CRS (days), *n* (range) | 2.0 (1–12) | | | 2.0 (1–13) | | | 2.0 (1–27) | | | 2.0 (1–8) | | | 2.0 (1–4) | | | 2.0 (1–3) | | |
| **ISS stage** | **I (*n =* 62)** | | **II (*n =* 53)** | | **III (*n =* 28)** | | **I (*n =* 64)** | | **II (*n =* 45)** | | **III (*n =* 35)** | | **I (*n =* 24)** | | **II (*n =* 18)** | | **III (*n =* 9)** | |
| Patients with CRS | 49 (79.0) | | 40 (75.5) | | 24 (85.7) | | 52 (81.3) | | 33 (73.3) | | 22 (62.9) | | 19 (79.2) | | 12 (66.7) | | 8 (88.9) | |
| Grade 1 | 43 (69.4) | | 32 (60.4) | | 14 (50.0) | | 44 (68.8) | | 24 (53.3) | | 15 (42.9) | | 15 (62.5) | | 10 (55.6) | | 2 (22.2) | |
| Grade 2 | 6 (9.7) | | 7 (13.2) | | 8 (28.6) | | 8 (12.5) | | 8 (17.8) | | 7 (20.0) | | 4 (16.7) | | 2 (11.1) | | 5 (55.6) | |
| Grade 3 | 0 | | 1 (1.9) | | 2 (7.1) | | 0 | | 1 (2.2) | | 0 | | 0 | | 0 | | 1 (11.1) | |
| Grade ≥4 | 0 | | 0 | | 0 | | 0 | | 0 | | 0 | | 0 | | 0 | | 0 | |
| Multiple CRS events | 19 (30.6) | | 17 (32.1) | | 10 (35.7) | | 24 (37.5) | | 14 (31.1) | | 8 (22.9) | | 7 (29.2) | | 1 (5.6) | | 5 (55.6) | |
| Median duration of CRS (days), *n* (range) | 2.0 (1–13) | | 2.0 (1–5) | | 2.0 (1–6) | | 2.0 (1–29) | | 2.0 (1–6) | | 2.0 (1–8) | | 2.0 (1–6) | | 2.0 (1–3) | | 2.0 (1–4) | |
| **EMD status** | **With EMD  (*n =* 33)** | | | **Without EMD (*n =* 110)** | | | **With EMD  (*n =* 37)** | | | **Without EMD (*n =* 108)** | | | **With EMD  (*n =* 16)** | | | **Without EMD  (*n =* 35)** | | |
| Patients with CRS | 27 (81.8) | | | 86 (78.2) | | | 29 (78.4) | | | 79 (73.1) | | | 13 (81.3) | | | 26 (74.3) | | |
| Grade 1 | 19 (57.6) | | | 70 (63.6) | | | 20 (54.1) | | | 63 (58.3) | | | 8 (50.0) | | | 19 (54.3) | | |
| Grade 2 | 6 (18.2) | | | 15 (13.6) | | | 8 (21.6) | | | 16 (14.8) | | | 5 (31.3) | | | 6 (17.1) | | |
| Grade 3 | 2 (6.1) | | | 1 (0.9) | | | 1 (2.7) | | | 0 | | | 0 | | | 1 (2.9) | | |
| Grade ≥4 | 0 | | | 0 | | | 0 | | | 0 | | | 0 | | | 0 | | |
| Multiple CRS events | 9 (27.3) | | | 37 (33.6) | | | 12 (32.4) | | | 34 (31.5) | | | 5 (31.3) | | | 8 (22.9) | | |
| Median duration of CRS (days), *n* (range) | 2.0 (1–5) | | | 2.0 (1–13) | | | 2.0 (1–29) | | | 2.0 (1–27) | | | 2.0 (1–6) | | | 2.0 (1–4) | | |
| **BMPCs** | **<5 (*n =* 54)** | **≥5 to ≤30  (*n =* 52)** | | **>30 to <60  (*n =* 15)** | | **≥60  (*n =* 17)** | **<5  (*n =* 37)** | **≥5 to ≤30  (*n =* 46)** | | **>30 to <60  (*n =* 26)** | | **≥60 (*n =* 32)** | **<5 (*n =* 17)** | **≥5 to ≤30 (*n =* 16)** | | **>30 to <60 (*n =* 6)** | | **≥60 (*n =* 8)** |
| Patients with CRS | 41 (75.9) | 40 (76.9) | | 14 (93.3) | | 14 (82.4) | 27 (73.0) | 35 (76.1) | | 18 (69.2) | | 24 (75.0) | 13 (76.5) | 14 (87.5) | | 3  (50.0) | | 6  (75.0) |
| Grade 1 | 36 (66.7) | 30 (57.7) | | 12 (80.0) | | 8  (47.1) | 21 (56.8) | 29 (63.0) | | 11 (42.3) | | 18 (56.3) | 9  (52.9) | 11 (68.8) | | 1  (16.7) | | 4  (50.0) |
| Grade 2 | 5 (9.3) | 9 (17.3) | | 1 (6.7) | | 6 (35.3) | 5 (13.5) | 6 (13.0) | | 7 (26.9) | | 6 (18.8) | 4 (23.5) | 3 (18.8) | | 2 (33.3) | | 1 (12.5) |
| Grade 3 | 0 | 1 (1.9) | | 1 (6.7) | | 0 | 1 (2.7) | 0 | | 0 | | 0 | 0 | 0 | | 0 | | 1 (12.5) |
| Grade ≥4 | 0 | 0 | | 0 | | 0 | 0 | 0 | | 0 | | 0 | 0 | 0 | | 0 | | 0 |
| Multiple CRS events | 15 (27.8) | 16 (30.8) | | 5  (33.3) | | 7  (41.2) | 11 (29.7) | 20 (43.5) | | 6  (23.1) | | 8  (25.0) | 4  (23.5) | 2  (12.5) | | 3  (50.0) | | 4  (50.0) |
| Median duration of CRS (days), *n* (range) | 2.0  (1–11) | 2.0  (1–12) | | 2.0  (1–13) | | 2.0  (1–5) | 2.0  (1–27) | 2.0  (1–29) | | 2.0  (1–8) | | 2.0  (1–14) | 2.0  (1–6) | 2.0  (1–4) | | 2.5  (1–4) | | 1.0  (1–2) |
| **Lymphocyte count** | **High^b^ (*n =* 66)** | | | **Low^c^ (*n =* 77)** | | | **High^b^ (*n =* 80)** | | | **Low^c^ (*n =* 64)** | | | **High^b^ (*n =* 23)** | | | **Low^c^ (*n =* 28)** | | |
| Patients with CRS | 52 (78.8) | | | 61 (79.2) | | | 57 (71.3) | | | 51 (79.7) | | | 20 (87.0) | | | 19 (67.9) | | |
| Grade 1 | 44 (66.7) | | | 45 (58.4) | | | 44 (55.0) | | | 39 (60.9) | | | 12 (52.2) | | | 15 (53.6) | | |
| Grade 2 | 8 (12.1) | | | 13 (16.9) | | | 13 (16.3) | | | 11 (17.2) | | | 7 (30.4) | | | 4 (14.3) | | |
| Grade 3 | 0 | | | 3 (3.9) | | | 0 | | | 1 (1.6) | | | 1 (4.3) | | | 0 | | |
| Grade ≥4 | 0 | | | 0 | | | 0 | | | 0 | | | 0 | | | 0 | | |
| Multiple CRS events | 18 (27.3) | | | 28 (36.4) | | | 26 (32.5) | | | 20 (31.3) | | | 6 (26.1) | | | 7 (25.0) | | |
| Median duration of CRS (days), *n* (range) | 2.0 (1–13) | | | 2.0 (1–12) | | | 2.0 (1–27) | | | 2.0 (1–29) | | | 2.0 (1–4) | | | 2.0 (1–6) | | |

Abbreviations: BMPC, bone marrow plasma cell; CRS, cytokine release syndrome; EMD, extramedullary disease; ISS, International Staging System; NR, not reported; QW, weekly; Q2W, every other week; SC, subcutaneous; TCRT, T-cell redirection therapy.

^a^Values are given as *n* (%) unless otherwise stated.

^b^High is ≥1000/mm^3^.

^c^Low is <1000/mm^3^.


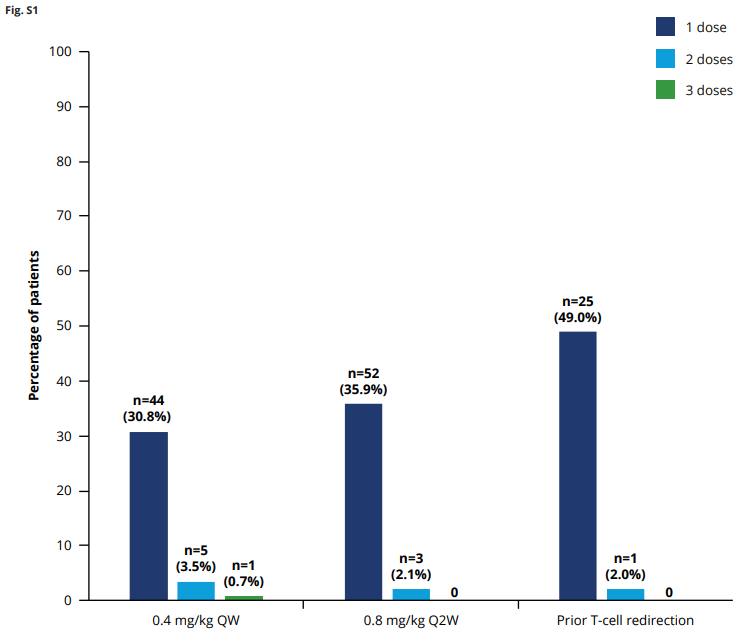


## SUPPLEMENTARY FIGURE S1 Percentage of patients who required one, two, or three doses of tocilizumab. QW indicates weekly; Q2W, every other week.
